# Cardiovascular Events 1 Year After Respiratory Syncytial Virus Infection in Adults

**DOI:** 10.1001/jamanetworkopen.2025.47618

**Published:** 2025-12-08

**Authors:** Anders Hviid, Thea K. Fischer, Tor Biering-Sørensen, Ingrid Bech Svalgaard

**Affiliations:** 1 Department of Epidemiology Research, Statens Serum Institut, Copenhagen, Denmark; 2 Department of Clinical Research, Nordsjællands Hospital, Hillerød, Denmark; 3 Department of Public Health, University of Copenhagen, Copenhagen, Denmark; 4 Center for Translational Cardiology and Pragmatic Randomized Trials, Department of Biomedical Sciences, Faculty of Health and Medical Sciences, University of Copenhagen, Copenhagen, Denmark; 5 Cardiovascular Non-Invasive Imaging Research Laboratory, Department of Cardiology, Copenhagen University Hospital–Herlev and Gentofte, Denmark; 6 Steno Diabetes Center, Copenhagen, Denmark; 7 Department of Cardiology, Copenhagen University Hospital–Rigshospitalet, Copenhagen, Denmark

## Abstract

**Importance:**

Respiratory syncytial virus (RSV) infection has recently been recognized as common among adults, but data on the burden of cardiovascular disease (CVD) beyond the immediate acute phase are lacking.

**Objective:**

To estimate the 365-day absolute excess risk (risk difference) of composites of CVD and their individual components following laboratory-confirmed RSV infection in adults.

**Design, Setting, and Participants:**

This nationwide, registry-based, matched cohort study was conducted using Danish national health registries. Adults aged 45 years or older on January 1, 2019, with a laboratory-confirmed RSV infection between January 1, 2019, and December 31, 2024, were matched 1:1 on age, sex, and preexisting comorbid conditions to individuals without RSV infection. Similar matched cohorts were established for influenza infection, hip fracture, and urinary tract infection without sepsis as control groups.

**Exposure:**

Laboratory-confirmed RSV infection.

**Main Outcomes and Measures:**

The primary outcomes were major adverse cardiovascular events, comprising ischemic heart disease, stroke, and heart failure, and any cardiovascular event, comprising major adverse cardiovascular events together with arrhythmias, venous thromboembolism, and inflammatory heart disease. Matched individuals were followed up for up to 365 days after the index date. Absolute risk differences and 95% CIs were calculated at 30 and 365 days using cumulative incidences derived from the Aalen-Johansen estimator.

**Results:**

The study included 17 494 matched individuals (mean [SD] age, 71.8 [12.0] years; 57.6% female). At 365 days, 665 any cardiovascular events had occurred among 8747 individuals with RSV infection and 257 among 8747 individuals without infection, corresponding to a risk difference of 4.69 percentage points (95% CI, 4.02-5.36 percentage points). The largest 365-day risk differences for any cardiovascular event were observed in hospitalized patients (6.61 percentage points [95% CI, 5.70-7.52 percentage points]), older individuals (eg, 7.93 percentage points [95% CI, 5.34-10.53 percentage points] for those aged 85-94 years), and preexisting CVD (11.95 percentage points [95% CI, 8.80-15.10 percentage points ]) or diabetes (7.50 percentage points [95% CI, 4.53-10.47 percentage points]). The 1-year cardiovascular event risk following RSV was comparable to that following influenza infection.

**Conclusions and Relevance:**

This cohort study of adults aged 45 years or older with RSV infection found a significant excess risk of cardiovascular events over 1 year, comparable in magnitude to influenza infection. These findings underscore the importance of RSV as a potential risk factor for cardiovascular morbidity and highlight the need for vaccination to mitigate this burden.

## Key Points

**Question:**

Is respiratory syncytial virus (RSV) infection in adults aged 45 years or older associated with an increased risk of cardiovascular events beyond the immediate acute phase?

**Findings:**

In this cohort study in 17 494 matched patients with and without RSV infection, RSV infection was associated with 4.69 additional cardiovascular events for every 100 older individuals with infection in the year following RSV infection.

**Meaning:**

These findings suggest that public health measures, such as vaccination, may prevent not only acute respiratory disease but also cardiovascular disease.

## Teaser

This cohort study estimates the 1-year absolute excess risk of any cardiovascular event following laboratory-confirmed respiratory syncytial virus (RSV) infection among adults in Denmark.

## Introduction

It is increasingly recognized that respiratory syncytial virus (RSV) infection is associated with a substantial burden of morbidity and mortality in older adults (aged ≥45 years), especially those with chronic underlying health conditions.^1,2^ In these vulnerable populations, RSV infection can lead to severe outcomes, including pneumonia, hospitalization, and death, with an overall disease burden that is comparable to that of seasonal influenza.^3,4^

Beyond the well-documented respiratory sequelae, current evidence supports that acute respiratory infections are strong, albeit transient, risk factors for cardiovascular disease (CVD). For instance, influenza infection has been consistently associated with an increased short-term risk of myocardial infarction and stroke.^5,6^ Similarly, there is a growing body of evidence suggesting that RSV infection may similarly act as an important trigger for acute CVD in adults.^7,8^

Despite this evidence, comprehensive data on the long-term clinical impact of various specific CVDs following RSV infection are scarce. Existing studies primarily reported on the immediate acute phase (eg, within 7-30 days after infection), with very few reporting on long-term impacts. A French study compared the risk of incident CVD among patients with RSV and influenza infection presenting at an emergency department and found no significant difference within 1 year.^9^ An Italian study followed up patients hospitalized for RSV for 1 year and reported an all-cause mortality of 29.6%, with 44.4% rehospitalized for any cause, 8.2% experiencing a stroke, and 3.3% experiencing an acute myocardial infarction.^10^

Other substantial gaps remain in understanding the full cardiovascular impact of RSV infections. Much of the current literature has focused on relative risk estimates, which while important for uncovering an association, do not fully capture the clinical burden. Studies describing the burden of cardiac events in adults with RSV infection often have methodological limitations that hinder a full understanding of the attributable risk and long-term cardiovascular outcomes of the infection. For instance, some studies may be cross-sectional in design, focusing solely on the prevalence of cardiac events during RSV-associated hospitalizations, or may lack a control group without infection. A notable example is a recent cross-sectional study that used data from the RSV Hospitalization Surveillance Network in the US, which found that 22.4% of hospitalized adults aged 50 years or older with RSV infection experienced an acute cardiac event, most frequently acute heart failure.^11^ The authors themselves noted key limitations, including the inability to associate observed acute cardiac events with the RSV infection due to the cross-sectional study design and lack of a control group.

Absolute risk differences directly quantify the number of excess events associated with an exposure within a defined population and time frame, both essential for clinical decision-making and public health policy guidance. With the recent licensure of 3 new RSV vaccines for use in older adults, understanding the precise magnitude of an RSV infection–associated cardiovascular burden has become even more critical.^12,13^ Such data are essential to accurately model the potential impact of vaccination programs on reducing cardiovascular morbidity and understanding the full spectrum of cost-effectiveness of these new vaccines.

Our aim in this study was to comprehensively quantify the absolute excess risks of a range of specific CVD events occurring up until 1 year after RSV infection among Danish adults. We also sought to contextualize the resulting absolute excess risks against those following influenza infection, hip fracture, and urinary tract infection without sepsis.

## Methods

### Study Design and Data Sources

This nationwide, register-based, matched cohort study leveraged Denmark’s comprehensive and linkable national health registries. The analyses were performed as surveillance activities as part of the advisory tasks of the governmental institution Statens Serum Institut (SSI) for the Danish Ministry of Health. The SSI’s purpose is to monitor and fight the spread of disease in accordance with section 222 of the Danish Health Act. Under Danish law, national surveillance activities conducted by SSI do not require approval and written informed consent from an ethics committee. The study followed the Strengthening the Reporting of Observational Studies in Epidemiology (STROBE) reporting guideline.

Denmark’s universal public health care system and the use of a unique personal identification number for all residents provide accurate linkage of individual-level data across various demographic characteristics and health registries.^14^ The Danish Civil Registration System has been providing daily updated information on vital status (death, emigration), residency, and demographic details for all Danish residents since 1968,^15^ allowing for the construction of a nationwide source cohort of every individual aged 45 years or older on January 1, 2019. Laboratory-confirmed RSV and influenza test results (all polymerase chain reaction [PCR] based) were available from the Danish Microbiology Database, a national database established in 2010 that contains data on all microbiological test results from all clinical microbiology departments in Denmark.^16^ Comorbidities and study outcomes were sourced from the Danish National Patient Registry, which contains records of all hospital contacts (inpatient, outpatient, and emergency department) with diagnoses coded according to the *International Statistical Classification of Diseases, Tenth Revision* (*ICD-10*).^17^

### Study Population and Matching

We identified all adults aged 45 years or older on January 1, 2019, who had resided in Denmark in the 5 years preceding January 1, 2019, and who had a first-time, laboratory-confirmed positive RSV PCR test recorded in the Danish Microbiology Database during the study period (January 1, 2019, to December 31, 2024). Individuals with a positive RSV test recorded in the 5 years preceding January 1, 2019, were excluded. Any potential subsequent RSV infections in a given individual during the study period were not included. Individuals with RSV infection were matched on the date of testing positive to individuals with no positive test recorded up until and on this day, 1:1, by exact matching without replacement on sex, age (5-year intervals), and the presence of the following comorbid conditions at the index date: asthma, an autoimmune disorder, CVD, chronic respiratory disorder, diabetes, kidney disorder, cancer, rheumatologic or inflammatory disorder, other intrinsic immune condition or immunodeficiency, and organ or stem cell transplant receipt. The index date for individuals in a matched pair was the date of the first positive RSV test for individuals with the infection. If an individual matching as not infected later tested positive for RSV after their assigned index date, follow-up for the matched pair was censored at that time. The individual newly infected could then reenter the study in a new matched pair on that given date provided that they could be successfully matched to another individual without infection.

### Exposure and Comparator Groups

The primary exposure group consisted of individuals with laboratory-confirmed RSV infection. The primary unexposed group comprised the matched individuals with no laboratory-confirmed RSV infection on or before their index date. Hospitalization for RSV was defined as a hospitalization with a laboratory-confirmed infection in the period 14 days before admission and until and including the day of admission. To provide clinical context, we also used 3 other comparison groups consisting of individuals with a first laboratory-confirmed influenza A/B infection (positive PCR test from the Danish Microbiology Database), an incident diagnosis of hip fracture, and an incident diagnosis of urinary tract infection without sepsis. These events provide a benchmark for the cardiovascular risk of RSV against other triggers, such as major trauma and severe bacterial infection. For all 3 of these outcomes, a history of the outcome before the study period started was allowed, but any further events during the study period were not included. To compare individuals with RSV infection with control individuals from these 3 comparison groups, 1:1 exact matching on age and sex and propensity score matching on all aforementioned comorbidities were performed while not allowing for self-matching. The date of the positive test (for influenza) or hospital admittance (for hip fracture or urinary tract infection without sepsis) was used as the index date for control individuals in the alternative comparison groups.

### Outcomes

Outcomes were identified through primary or secondary (A or B) *ICD-10* discharge diagnoses recorded in the Danish National Patient Registry from any hospital contact (inpatient admission, outpatient contact, or emergency department visit) lasting at least 24 hours. The contact start date was considered the time of the event. We included the following subgroups of CVD: ischemic heart disease, stroke, venous thromboembolism, heart failure, arrhythmias, and inflammatory heart disease (carditis). We used the following composite measures: major adverse cardiovascular events (MACEs) composed of ischemic heart disease, stroke, and heart failure and any cardiovascular event composed of MACEs, venous thromboembolism, arrhythmias, and inflammatory heart disease. Specific *ICD-10* codes are presented in eTable 1 in Supplement 1. An individual could contribute to multiple specific outcome categories if they experienced distinct events. Outcome hospitalizations with admission dates before the test date were not attributed to RSV in the event that a positive test was obtained during the hospital stay. Outcome admissions after a positive test, including same-day admissions, were counted as outcomes among individuals with RSV infection. In analyses of single outcomes, other outcomes than the one under study did not censor follow-up.

### Covariates

Information on baseline comorbidities was extracted from national registries. Age was calculated at the index date. Sex was obtained from the Civil Registration System. Preexisting comorbidities were identified based on relevant *ICD-10* hospital discharge diagnose codes recorded in the Danish National Patient Registry within a historic 5-year period prior to the index date. Detailed definitions and *ICD-10* codes for comorbidities are provided in eTable 1 in Supplement 1.

### Statistical Analysis

Baseline characteristics of individuals with RSV infection and matched individuals without infection are summarized using counts and percentages for categorical variables. Exact matching ensured that individuals with and without infection were perfectly balanced on the included covariates.

Matched pairs were followed up for 365 days after the index date. A matched pair was censored if (1) the control individual had a positive laboratory test for RSV; (2) the end of the study period on December 31, 2024; or (3) death or emigration.

During follow-up, we estimated the cumulative incidence of each CVD outcome during the 365 days after the index date. Cumulative incidences were estimated using the Aalen-Johansen estimator, which accounts for the competing risk of death. Relative risks and absolute risk differences (in percentage points) at 30 and 365 days were calculated using the cumulative incidences in the exposed and unexposed groups, with 95% CIs constructed using the delta method. Estimates were considered statistically significant if the 95% CI did not contain 0 (for risk differences) or 1 (for risk ratios).

Primary analyses compared outcomes between individuals with RSV infection and their matched counterparts without infection. Secondary analyses involved similar comparisons between individuals with RSV infection and control individuals with influenza, hip fracture, and urinary tract infection, using the any cardiovascular event composite outcome. Hip fracture was also used as a negative control outcome in the RSV infection vs no infection comparison.

Prespecified subgroup analyses were performed for the primary RSV infection vs no infection comparison to explore heterogeneity of the burden. These subgroups were defined by age categories; sex; calendar period; and presence of preexisting CVD, diabetes, or immunocompromise at the index date. Data management and statistical analyses were performed using R, version 4.1.1 (R Foundation for Statistical Computing) with the packages MatchIt and survival.

## Results

The source cohort comprised 2 622 667 individuals with at least 5 years of residence in Denmark and who were aged 45 years or older at the start of the study period. In the source cohort, 8825 had a first-time recording of laboratory-confirmed RSV infection, of whom 8747 (99.1%) were successfully matched 1:1 to 8747 individuals without infection, resulting in a total study cohort of 17 494 participants (mean [SD] age, 71.8 [12.0] years; 57.6% female and 42.4% male) (eFigure 1 in Supplement 1). The distribution of RSV infections over time showed the suppression of events in the 2020 to 2021 winter season and a rebound afterward, with a shift toward an earlier start of the RSV season (eFigure 2 in Supplement 1).

In the matched cohort, 41 individuals (0.5%) transitioned from no infection to RSV infection, corresponding to 1.4 events per 100 000 person-days. The baseline characteristics of the RSV-infected and uninfected groups were well-balanced after matching (Table). A notable proportion, 11.5% (n = 2008), had a history of any CVD at baseline. Other common comorbidities included chronic respiratory disease (n = 2880 [16.5%]), a history of cancer (n = 3382 [19.3%]), and diabetes (n = 1480 [8.5%]). A total of 6045 individuals with RSV infection (69.1%) were hospitalized. Among the individuals without infection, 1415 (16.2%) had a hospital contact during the 14-day period preceding or including the index date.

**Table.** Baseline Characteristics of Matched Individuals With and Without RSV Infection

Characteristic	Individuals, No. (%)	
	RSV infection (n = 8747)	No RSV infection (n = 8747)
Sex		
Female	5039 (57.6)	5039 (57.6)
Male	3708 (42.4)	3708 (42.4)
Age, mean (SD), y	71.89 (12.00)	71.82 (12.00)
Preexisting comorbid condition		
Asthma	617 (7.1)	617 (7.1)
Autoimmune disorder	275 (3.1)	275 (3.1)
Cardiovascular disease	1004 (11.5)	1004 (11.5)
Chronic respiratory disease	1440 (16.5)	1440 (16.5)
Diabetes	740 (8.5)	740 (8.5)
Kidney disorder	601 (6.9)	601 (6.9)
Cancer	1691 (19.3)	1691 (19.3)
Rheumatologic or inflammatory disorder	600 (6.9)	600 (6.9)
Other intrinsic immune condition or immunodeficiency	246 (2.8)	246 (2.8)
Organ or stem cell transplant receipt	278 (3.2)	278 (3.2)
Hospital contact in the 14 d preceding index date	6045 (69.1)	1415 (16.2)

Abbreviation: RSV, respiratory syncytial virus.

**Figure 1.**
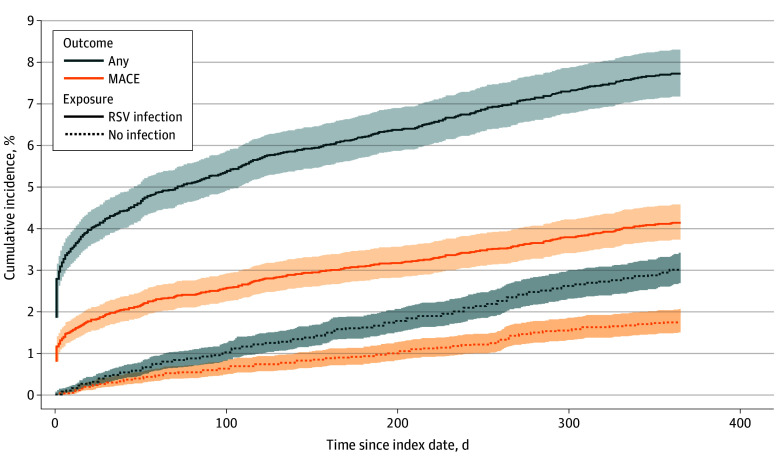
Cumulative Incidences of Any Cardiovascular Event and Major Adverse Cardiovascular Event (MACE) Among Individuals With and Without Respiratory Syncytial Virus (RSV) Infection

**Figure 2.**
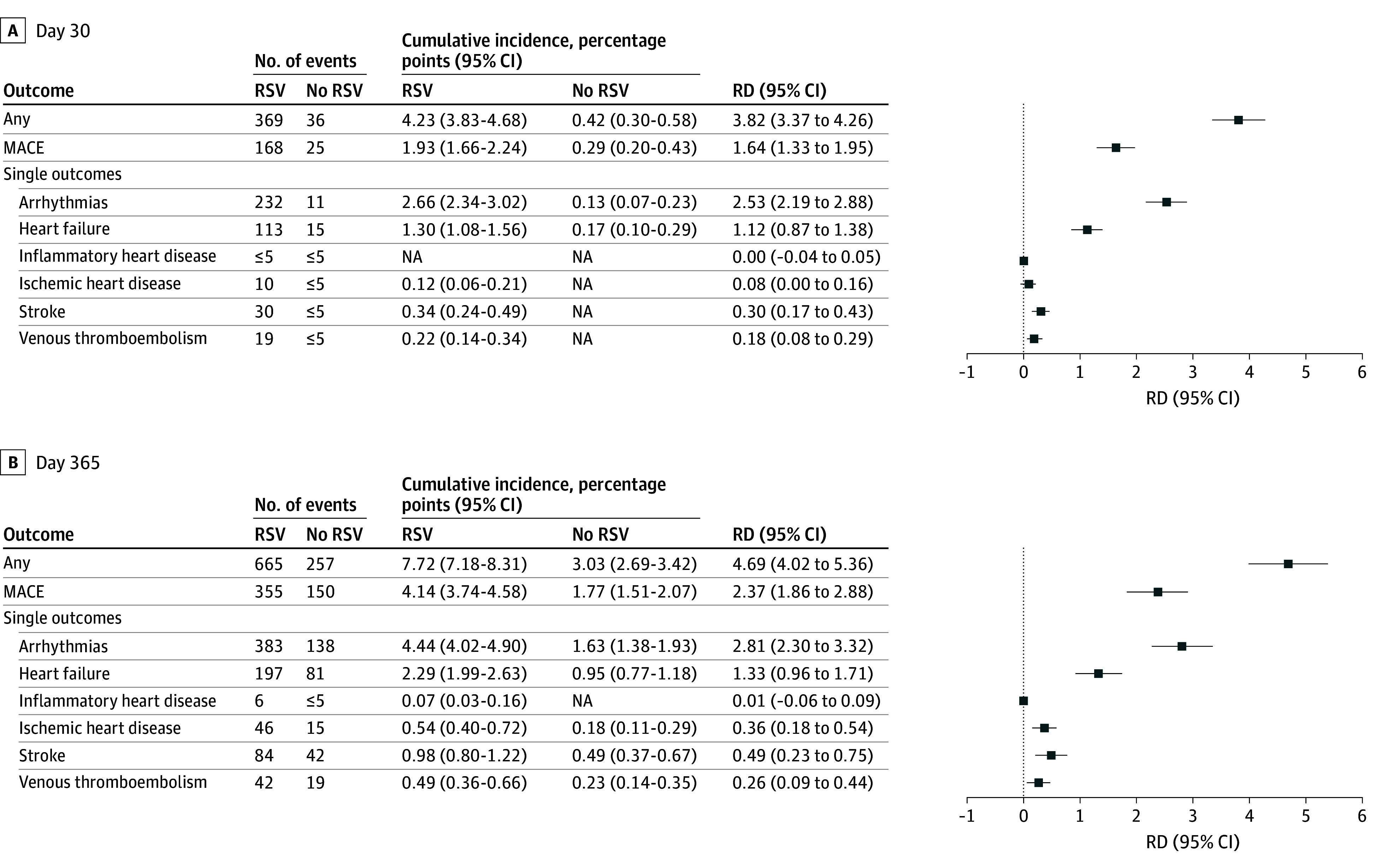
Risk Differences (RDs) of Any Cardiovascular Event Between Individuals With and Without Respiratory Syncytial Virus (RSV) Infection at 30 Days and 1 Year MACE indicates major adverse cardiovascular event; NA, not applicable due to a small number of events.

The cumulative incidences of any cardiovascular event and MACE were increased among individuals with RSV infection compared with those without infection throughout the 365 days of follow-up (Figure 1; eTable 2 in Supplement 1). A total of 665 and 257 any cardiovascular events occurred among individuals with and without RSV infection, respectively. These increases corresponded to 30-day risk differences of 3.82 percentage points (95% CI, 3.37-4.26 percentage points) and 1.64 percentage points (95% CI, 1.33-1.95 percentage points) for any cardiovascular event and MACE, respectively (Figure 2). At 365 days of follow-up, we observed further increased risk differences of 4.69 percentage points (95% CI, 4.02-5.36 percentage points) and 2.37 percentage points (95% CI, 1.86-2.88 percentage points), respectively. Infection with RSV was associated with increases in risk differences at both day 30 and day 365 for all single outcomes but inflammatory heart disease (30 days: risk difference, 0 percentage points [95% CI, −0.04 to 0.05]; 365 days: risk difference, 0.01 percentage points [95% CI, −0.06 to 0.09 percentage points]). Arrhythmias and heart failure were associated with the largest risk differences at both day 30 (arrhythmias: 2.53 percentage points [95% CI, 2.19-2.88 percentage points]; heart failure: 1.12 percentage points [95% CI, 0.87-1.38 percentage points]) and day 365 (arrhythmias: 2.81 percentage points [95% CI, 2.30-3.32 percentage points]; heart failure: 1.33 percentage points [95% CI, 0.96-1.71 percentage points]). The corresponding relative risks are presented in eFigure 3 in Supplement 1.

**Figure 3.**
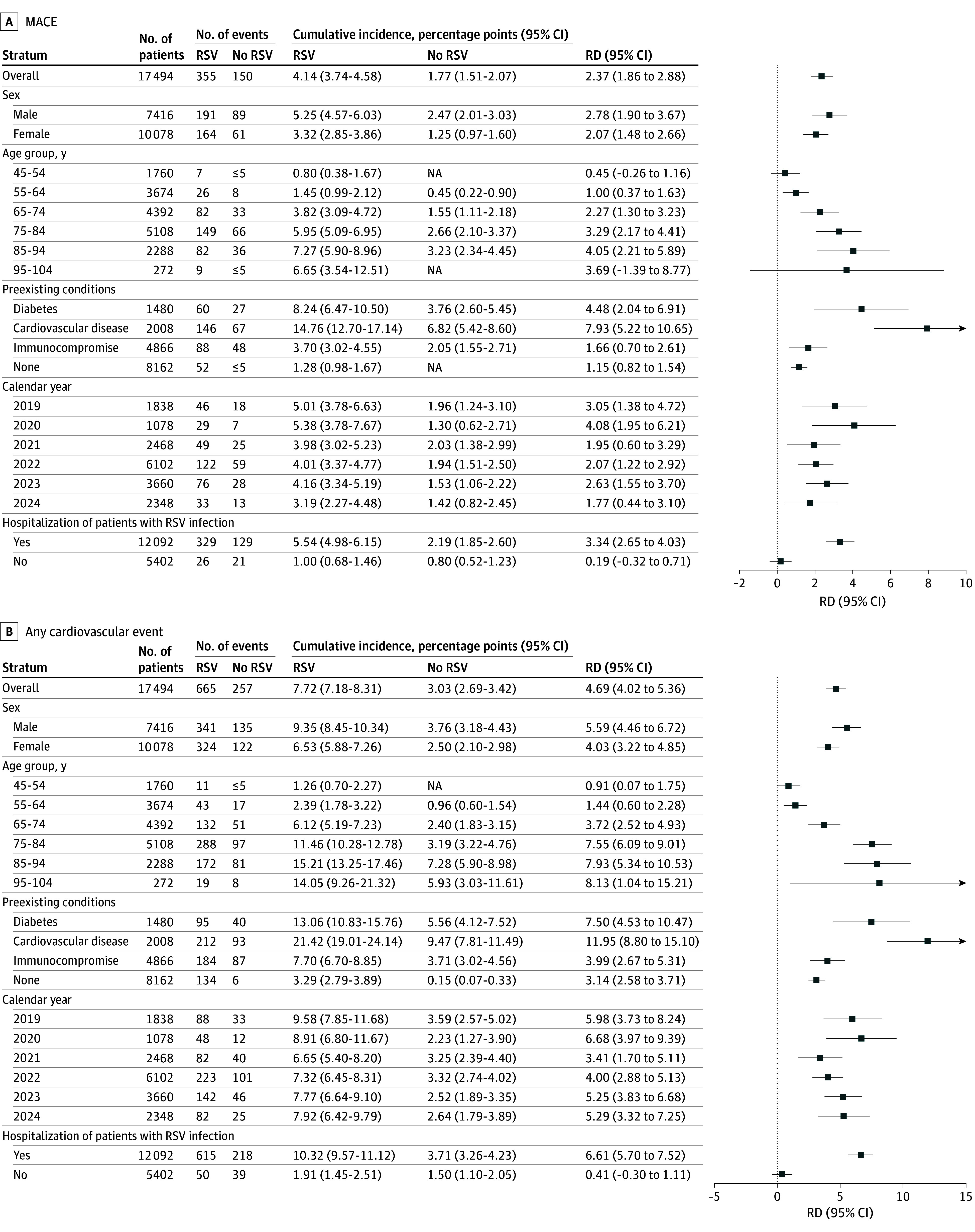
Subgroup Risk Differences (RDs) for Cardiovascular Events Between Individuals With and Without Respiratory Syncytial Virus (RSV) Infection at 1 Year MACE indicates major adverse cardiovascular event; NA, not applicable due to a small number of events.

Among the hospitalized patients, the 365-day risk differences were 6.61 percentage points (95% CI, 5.70-7.52 percentage points) for any cardiovascular event and 3.34 percentage points (95% CI, 2.65-4.03 percentage points) for MACE. Patients not hospitalized did not experience excess risk of either any cardiovascular event or MACE (Figure 3).

We observed heterogeneity in the 365-day risk differences across prespecified subgroups for both any cardiovascular event and MACE (Figure 3). Age was associated with the highest degree of heterogeneity. The 365-day risk difference for any cardiovascular event was 0.91 percentage points (95% CI, 0.07-1.75 percentage points) among patients aged 45 to 54 years compared with 7.93 percentage points (95% CI, 5.34-10.53 percentage points) among those aged 85 to 94 years. Preexisting CVD (any cardiovascular event: 365-day risk difference, 11.95 percentage points [95% CI, 8.80-15.10 percentage points]; diabetes: 365-day risk difference, 7.50 percentage points [95% CI, 4.53-10.47 percentage points]) were associated with the greatest burdens of cardiovascular events from RSV infection throughout the follow-up. For comparison, the corresponding risk difference for individuals without any preexisting comorbidities was 3.14 percentage points (95% CI, 2.58-3.71 percentage points). Risk differences across calendar periods and sex were less pronounced.

**Figure 4.**
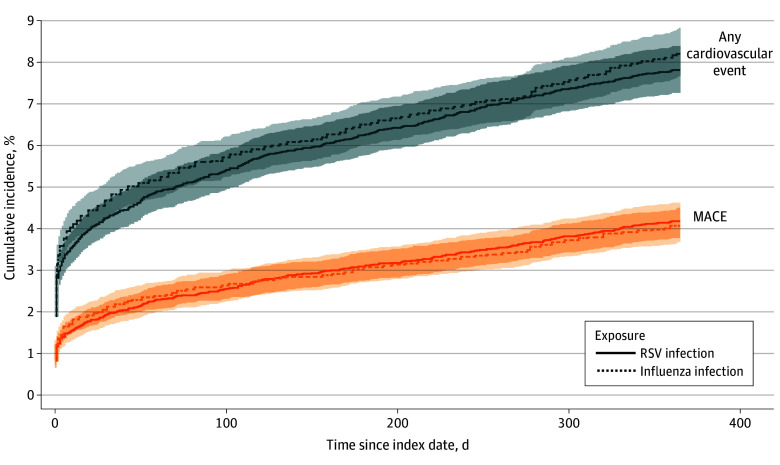
Cumulative Incidences of Any Cardiovascular Event and Major Adverse Cardiovascular Event (MACE) Between Individuals With Respiratory Syncytial Virus (RSV) Infection and Those With Influenza Infection

A total of 8823 individuals with laboratory-confirmed RSV infection were matched 1:1 to 8823 individuals with laboratory-confirmed influenza infection. Comparing these groups, we observed no significant differences in the cumulative incidences of any cardiovascular event and MACE throughout the follow-up (Figure 4; eTable 3 in Supplement 1). At day 30, the any cardiovascular event risk difference was −0.36 percentage points (95% CI, −0.97 to 0.24 percentage points), and at day 365, it was −0.42 percentage points (95% CI, −1.23 to 0.39 percentage points).

Among 8422 matched pairs of individuals with RSV infection and individuals with hip fracture, we observed differences in cumulative incidences of any cardiovascular event and MACE in the first 180 days of follow-up (eFigure 4 and eTable 4 in Supplement 1). The differences were most marked in the acute period, with 30-day risk differences of −1.97 percentage points (95% CI, −2.65 to −1.28 percentage points) and −1.09 percentage points (95% CI, −1.5 to −0.61 percentage points) for any cardiovascular event and MACE, respectively. At 365 days, the differences were less pronounced, with risk differences of −0.98 percentage points (95% CI, −1.84 to −0.13 percentage points) and −0.62 percentage points (95% CI, −1.27 to 0.03 percentage points) for any cardiovascular event and MACE, respectively.

Comparing 8825 individuals with RSV infection with matched individuals with urinary tract infection without sepsis, we observed differences in cumulative incidences of any cardiovascular event and MACEs throughout follow-up (eFigure 5 and eTable 5 in Supplement 1). On day 30, the risk differences were −1.39 percentage points (95% CI, −2.04 to −0.75 percentage points) and −0.81 percentage points (95% CI, −1.26 to −0.37 percentage points) for any cardiovascular event and MACE, respectively. At 365 days, we observed similar risk differences of −1.80 percentage points (95% CI, −2.65 to −0.96 percentage points) and −1.02 percentage points (95% CI, −1.65 to −0.39 percentage points) for any cardiovascular event and MACE, respectively. Using hip fracture as a negative control outcome, we observed no association with RSV infection, with a 30-day risk difference of 0.02 percentage points (95% CI, −0.04 to 0.09 percentage points) and a 365-day risk difference of 0.14 percentage points (95% CI, −0.05 to 0.33 percentage points).

## Discussion

This comprehensive, nationwide, matched cohort study of Danish adults estimated the 1-year absolute excess risk of a wide range of cardiovascular events following laboratory-confirmed RSV infection. We found that RSV infection is associated with a statistically significant and clinically meaningful increase in the absolute risk of cardiovascular events, also beyond the acute period, comparable to that of influenza infection. The burden on day 365 of follow-up corresponded to 4.69 additional cardiovascular events per 100 individuals with RSV infection compared with those without infection. The majority of this excess risk was apparent within the first 30 days, but the cumulative burden continued to accrue over the subsequent year. Hospitalization, older age, and preexisting CVD and diabetes were associated with the largest burdens, but younger individuals and individuals without comorbidities still experienced outcomes, though less so.

Few studies have evaluated the cardiovascular burden of RSV infection. In an Italian cohort of 243 hospitalized older adults with RSV infection and no control group, high 1-year incidence rates of stroke (8.2%) and acute myocardial infarction (3.3%) were observed together with 29.6% all-cause mortality.^10^ Despite the majority of infections (69.1%) in our study also resulting in hospitalizations and similar mean ages (72.7 years in the Italian study vs 71.8 years in our study), our findings suggest a lower burden of stroke and ischemic heart disease than in the Italian hospitalized population (0.49 and 0.36 additional cases of stroke and ischemic heart disease for 100 individuals with RSV infection vs without infection). In a larger US cohort of 6248 hospitalizations for RSV infection (median [IQR] age, 72.7 [63.0-82.3] years), 22.4% of patients experienced an acute cardiac event, most often heart failure (15.8%).^11^ This proportion is also many more than the 1.12 additional cases of heart failure we observed in our 30-day acute period, reflecting differences in thresholds for hospitalization and RSV testing of adults^18^ in the different countries, as well as differences in which and how cardiovascular events were coded and the lack of control groups. Our finding of comparable 1-year cardiovascular event rates between RSV and influenza infection is consistent with observations from French emergency department patients.^9^ Among 3224 patients, no significant differences in odds of adverse cardiac events within 1 year were observed comparing patients with influenza vs RSV infection (odds ratio, 1.36 [95% CI, 0.72-2.57]). This risk is noteworthy, as some studies have reported greater RSV-associated morbidity and/or mortality compared with influenza, but differences in indication for testing may be a contributing factor.^4,19^

The findings of a significant and year-long cardiovascular burden associated with RSV infection, comparable to that of influenza infection, underscores the potential for an important public health impact through RSV vaccination programs in vulnerable populations, such as older adults, which may not only reduce respiratory illness but also play a crucial role in mitigating a considerable burden of subsequent CVD. Future research should evaluate the impact of RSV vaccination on cardiovascular outcomes and further explore the mechanisms underlying this prolonged risk.

### Strengths and Limitations

Key strengths of our study included its nationwide, registry-based design using comprehensive Danish health data, ensuring a large, representative cohort with individual-level linkage. The 1:1 matching on age, sex, and an extensive list of comorbid conditions enabled robust estimation of the absolute excess risk associated with RSV infection in the exposed population.

The study also had some limitations. While the nationwide design supports generalizability to other high-income countries, our specific risk estimates are based on Denmark’s homogeneous population and universal health care system and may not be directly transferable to countries with more heterogeneous populations and other health care models. Other limitations include the possibility of unmeasured confounding, eg, through lifestyle factors or sociodemographics, and the potential for underascertainment of RSV infection in the control group. Unrecognized infections in the control group would tend to attenuate the risk differences.

## Conclusions

This nationwide cohort study found that RSV infection in older adults is associated with a significant and year-long cumulative absolute excess risk for a range of specific and serious cardiovascular events. The magnitude of these risk differences, which are comparable to those observed following influenza infection, highlights the substantial and prolonged cardiovascular burden imposed by RSV infection. These findings underscore the importance of recognizing RSV not only as a respiratory pathogen but also as an important risk factor for cardiovascular morbidity in adults. This study should prompt heightened clinical vigilance for cardiovascular complications following RSV infection and reinforces the public health rationale for preventive measures, most notably vaccination, in older adult populations to mitigate this burden.

## Notes

Supplement 1. **eTable 1.** *International Statistical Classification, Tenth Revision* (*ICD-10*) Codes for Study Outcomes, Negative Control Outcomes, Secondary Comparator Groups, and Comorbidities **eFigure 1.** Study Population Flowchart **eFigure 2.** Distribution of Individuals With Laboratory-Confirmed RSV Infection Over Time **eFigure 3. **30-Day and 365-Day Cardiovascular Event Risk Ratios (RRs) Comparing Individuals With and Without RSV Infection **eTable 2. **Number of Individuals at Risk and Number of Any Cardiovascular Events and MACEs at Selected Time Points Among Individuals With and Without RSV Infection **eTable 3. **Number of Individuals at Risk and Number of Any Cardiovascular Events and MACEs at Selected Time Points Among Individuals With RSV Infection and Individuals With Influenza Infection **eFigure 4.** Cumulative Incidences of Any Cardiovascular Event and MACE, Respectively, Among Individuals With RSV Infection and Individuals With Hip Fracture **eTable 4.** Number of Individuals at Risk and Number of Any Cardiovascular Events and MACEs at Selected Time Points Among Individuals With RSV Infection and Individuals With Hip Fracture **eFigure 5.** Cumulative Incidences of Any Cardiovascular Event and MACE, Respectively, Among Individuals With RSV Infection and Individuals With Urinary Tract Infection Without Sepsis **eTable 5.** Number of Individuals at Risk and Number of Any Cardiovascular Events and MACEs at Selected Time Points Among Individuals With RSV Infection and Individuals With Urinary Tract Infection Without Sepsis

Supplement 2.
Data Sharing Statement
