# Supplementary material for: Cardiovascular Events 1 Year After Respiratory Syncytial Virus Infection in Adults
Source: JAMA Netw Open. 2025 Dec 8;8(12):e2547618. doi: 10.1001/jamanetworkopen.2025.47618 (PMC12687100; doi:10.1001/jamanetworkopen.2025.47618)
Supplement: Supplement 1. — eTable 1. International Statistical Classification, Tenth Revision (ICD-10) Codes for Study Outcomes, Negative Control Outcomes, Secondary Comparator Groups, and Comorbidities eFigure 1. Study Population Flowchart eFigure 2. Distribution of Individuals With Laboratory-Confirmed RSV Infection Over Time eFigure 3. 30-Day and 365-Day Cardiovascular Event Risk Ratios (RRs) Comparing Individuals With and Without RSV Infection eTable 2. Number of Individuals at Risk and Number of Any Cardiovascular Events and MACEs at Selected Time Points Among Individuals With and Without RSV Infection eTable 3. Number of Individuals at Risk and Number of Any Cardiovascular Events and MACEs at Selected Time Points Among Individuals With RSV Infection and Individuals With Influenza Infection eFigure 4. Cumulative Incidences of Any Cardiovascular Event and MACE, Respectively, Among Individuals With RSV Infection and Individuals With Hip Fracture eTable 4. Number of Individuals at Risk and Number of Any Cardiovascular Events and MACEs at Selected Time Points Among Individuals With RSV Infection and Individuals With Hip Fracture eFigure 5. Cumulative Incidences of Any Cardiovascular Event and MACE, Respectively, Among Individuals With RSV Infection and Individuals With Urinary Tract Infection Without Sepsis eTable 5. Number of Individuals at Risk and Number of Any Cardiovascular Events and MACEs at Selected Time Points Among Individuals With RSV Infection and Individuals With Urinary Tract Infection Without Sepsis [file jamanetwopen-e2547618-s001.pdf]

Supplemental Online Content

Hviid A, Fischer TK, Biering-Sørensen T, Bech Svalgaard I. Cardiovascular events 1 year after respiratory syncytial virus infection in adults. *JAMA Netw Open*. 2025;8(11):e2547618. doi:10.1001/jamanetworkopen.2025.47618

eTable 1. *International Statistical Classification, Tenth Revision* (*ICD-10*) Codes for Study Outcomes, Negative Control Outcomes, Secondary Comparator Groups, and Comorbidities
eFigure 1. Study Population Flowchart
eFigure 2. Distribution of Individuals With Laboratory-Confirmed RSV Infection Over Time
eFigure 3. 30-Day and 365-Day Cardiovascular Event Risk Ratios (RRs) Comparing Individuals With and Without RSV Infection
eTable 2. Number of Individuals at Risk and Number of Any Cardiovascular Events and MACEs at Selected Time Points Among Individuals With and Without RSV Infection
eTable 3. Number of Individuals at Risk and Number of Any Cardiovascular Events and MACEs at Selected Time Points Among Individuals With RSV Infection and Individuals With Influenza Infection
eFigure 4. Cumulative Incidences of Any Cardiovascular Event and MACE, Respectively, Among Individuals With RSV Infection and Individuals With Hip Fracture
eTable 4. Number of Individuals at Risk and Number of Any Cardiovascular Events and MACEs at Selected Time Points Among Individuals With RSV Infection and Individuals With Hip Fracture
eFigure 5. Cumulative Incidences of Any Cardiovascular Event and MACE, Respectively, Among Individuals With RSV Infection and Individuals With Urinary Tract Infection Without Sepsis
eTable 5. Number of Individuals at Risk and Number of Any Cardiovascular Events and MACEs at Selected Time Points Among Individuals With RSV Infection and Individuals With Urinary Tract Infection Without Sepsis

This supplemental material has been provided by the authors to give readers additional information about their work.

© 2025 Hviid A et al. *JAMA Network Open*.

eTable 1. *International Statistical Classification, Tenth Revision* (*ICD-10*) Codes for Study Outcomes, Negative Control Outcomes, Secondary Comparator Groups, and Comorbidities

**Condition**	***ICD-10* code(s)**
**Study Outcomes**	
Ischaemic Heart Disease	I20.0, I21, I22, I24.0, I24.8, I24.9.
Stroke	I60, I61, I62, I63, I64.
Venous Thromboembolism	I26, I80.1, I80.2, I80.3, I80.8, I80.9, I81, I82.
Heart Failure	I50.0, I50.1, I50.9.
Arrhythmias	I47, I48, I49.
Inflammatory heart disease	I30, I33, I40.
**Negative Control Outcome**	
Hip Fracture	S72.0, S72.1, S72.2.
**Comparator Groups**	
Hip Fracture	S72.0, S72.1, S72.2.
Urinary Tract Infection without Sepsis	N10, N30.0, N30.8, N30.9, N39.0.<br>Exclusion Codes (30 days before until 30 days after UTI):<br>A40, A41, R57.2, N17.
**Comorbidities**	
Asthma	J45, J46
Autoimmune disorder	D510, D590, D591, D690, D693, D86, E050, E063, E271, E272, G122G, G35, G610, G700, I00, I01, K50, K51, K743, K900, L12, L40, L52, L80, L93, M05, M06, M08, M300, M313, M315, M316, M32, M33, M34, M35, M45.
Cardiovascular disease	I110, I130, I132, I20-I23, I420, I426-I429, I48, I500-I503, I508, I509.
Chronic respiratory disorder	J40-J47, J60-J67, J684, J701, J703, J841, J920, J961, J982, J983.
Diabetes	E10-E11.

© 2025 Hviid A et al. *JAMA Network Open*.

eTable 1. *International Statistical Classification, Tenth Revision* (*ICD-10*) Codes for Study Outcomes, Negative Control Outcomes, Secondary Comparator Groups, and Comorbidities	
Renal disorder	I12, I13, N00-N05, N07, N11, N14, N17-N19, Q61.
Malignancy	C00-C85 (without C44), C88, C90-C96.
Rheumatologic or inflammatory disorder	D86, E85 (without E850), G35, J679, L401, L405, L93, L94, M05, M06, M07, M08, M30, M313, M315, M32-M35, M46
Other intrinsic immune condition or immunodeficiency	B9735, C279, D61, D728, D80, D81 (without D813), D82-D84, D89 (without D892, K704, K72, K743-K746 (without K7460, K7469), N04, R18, Z992
Organ or stem cell transplant recipient	T86 (without 8682–T8684, T8689, and T869), Z94, Z9885

© 2025 Hviid A et al. *JAMA Network Open*.

eFigure 1. Study Population Flowchart. The diagram illustrates the selection of the study cohort, matching process, follow-up period, and outcomes assessed. Abbreviations: CVD, cardiovascular disease; IHD, ischemic heart disease; MACE, major adverse cardiovascular events; PCR+, polymerase chain reaction positive; RSV, respiratory syncytial virus; VTE, venous thromboembolism.

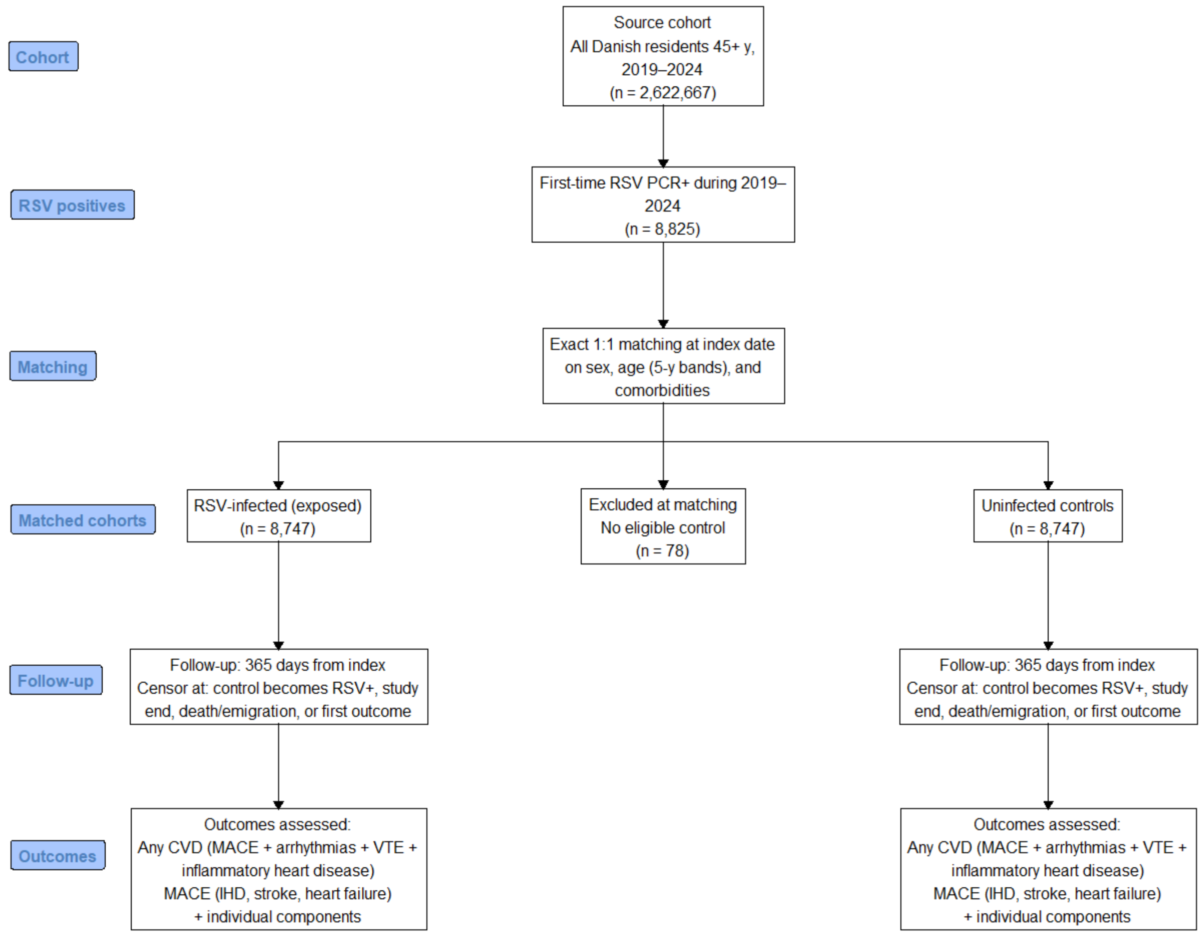

© 2025 Hviid A et al. *JAMA Network Open*.

eFigure 2. Distribution of Individuals With Laboratory-Confirmed RSV Infection Over Time

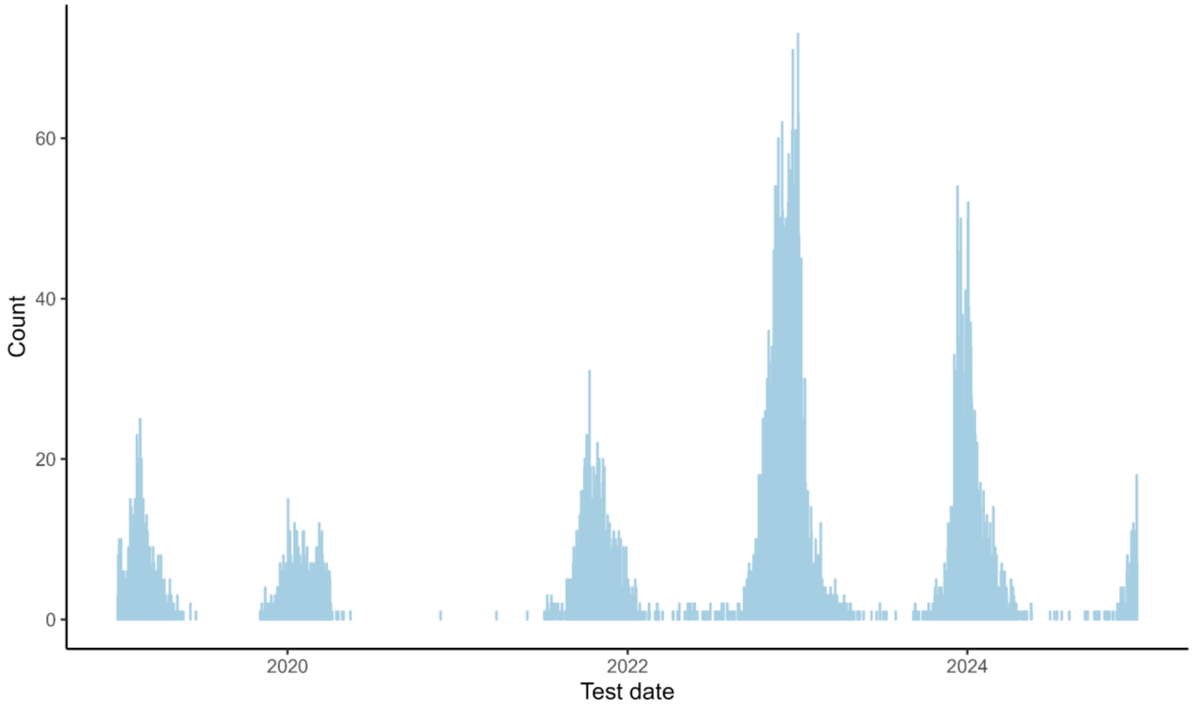

© 2025 Hviid A et al. *JAMA Network Open*.

eFigure 3. 30-Day and 365-Day Cardiovascular Event Risk Ratios (RRs) Comparing Individuals With and Without RSV Infection

	Day 30					Day 365				
	No. of events		Cumulative incidence (95% CI)			No. of events		Cumulative incidence (95% CI)		
**Outcome**	**RSV**	**No RSV**	**RSV**	**No RSV**	**RR (95% CI)**	**RSV**	**No RSV**	**RSV**	**No RSV**	**RR (95% CI)**
Any	369	36	4.23 (3.83, 4.68)	0.42 (0.3, 0.58)	10.17 (9.15, 11.18)	665	257	7.72 (7.18, 8.31)	3.03 (2.69, 3.42)	2.55 (2.36, 2.73)
MACE	168	25	1.93 (1.66, 2.24)	0.29 (0.2, 0.43)	6.66 (5.66, 7.66)	355	150	4.14 (3.74, 4.58)	1.77 (1.51, 2.07)	2.34 (2.1, 2.58)
**Single outcomes**										
Arrhythmias	232	11	2.66 (2.34, 3.02)	0.13 (0.07, 0.23)	20.9 (18.25, 23.56)	383	138	4.44 (4.02, 4.9)	1.63 (1.38, 1.93)	2.72 (2.45, 2.99)
Heart failure	113	15	1.3 (1.08, 1.56)	0.17 (0.1, 0.29)	7.47 (6.1, 8.83)	197	81	2.29 (1.99, 2.63)	0.95 (0.77, 1.18)	2.4 (2.07, 2.73)
Inflammatory heart disease	≤5	≤5	Not shown	Not shown	1 (-0.39, 2.4)	6	≤5	0.07 (0.03, 0.16)	Not shown	1.2 (0.24, 2.15)
Ischemic heart disease	10	≤5	0.12 (0.06, 0.21)	Not shown	3.31 (1.26, 5.35)	46	15	0.54 (0.4, 0.72)	0.18 (0.11, 0.29)	3.07 (2.18, 3.95)
Stroke	30	≤5	0.34 (0.24, 0.49)	Not shown	7.46 (4.79, 10.12)	84	42	0.98 (0.8, 1.22)	0.49 (0.37, 0.67)	1.99 (1.57, 2.41)
Venous thromboembolism	19	≤5	0.22 (0.14, 0.34)	Not shown	6.25 (3.44, 9.05)	42	19	0.49 (0.36, 0.66)	0.23 (0.14, 0.35)	2.17 (1.52, 2.83)

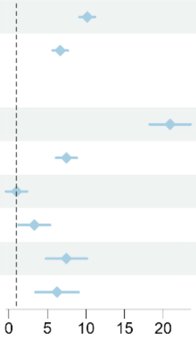

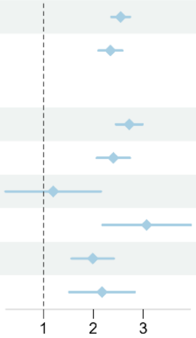

© 2025 Hviid A et al. *JAMA Network Open*.

eTable 2. Number of Individuals at Risk and Number of Any Cardiovascular Events and MACEs at Selected Time Points Among Individuals With and Without RSV Infection

			Time (days)						
			0	10	20	30	100	200	365
Any	RSV infected	*Events*	164	146	37	23	97	84	114
		At risk	8747	8042	7831	7719	7363	7056	5918
	Uninfected	*Events*	≤12	≤12	14	10	52	64	105
		At risk	8747	8634	8548	8494	8307	8128	6927
MACE	RSV infected	*Events*	72	64	19	14	54	51	81
		At risk	8747	8201	8001	7895	7566	7271	6103
	Uninfected	*Events*	0	6	11	8	31	33	61
		At risk	8747	8640	8556	8504	8330	8170	6992

eTable 3. Number of Individuals at Risk and Number of Any Cardiovascular Events and MACEs at Selected Time Points Among Individuals With RSV Infection and Individuals With Influenza Infection

			Time (days)						
			0	10	20	30	100	200	365
Any	RSV	*Events*	168	146	37	24	99	87	118
		At risk	8823	8111	7899	7782	7426	7112	5963
	Influenza	*Events*	200	161	29	20	90	79	130
		At risk	8823	8094	7884	7782	7438	7145	5419
MACE	RSV	*Events*	73	64	19	14	54	53	85
		At risk	8747	8201	8001	7895	7566	7271	6103
	Influenza	*Events*	78	76	16	12	48	43	77
		At risk	8823	8286	8086	7985	7669	7387	5642

© 2025 Hviid A et al. *JAMA Network Open*.

Figure S4: Cumulative Incidences of Any Cardiovascular Event and MACE, Respectively, Among Individuals With RSV Infection and Individuals With Hip Fracture

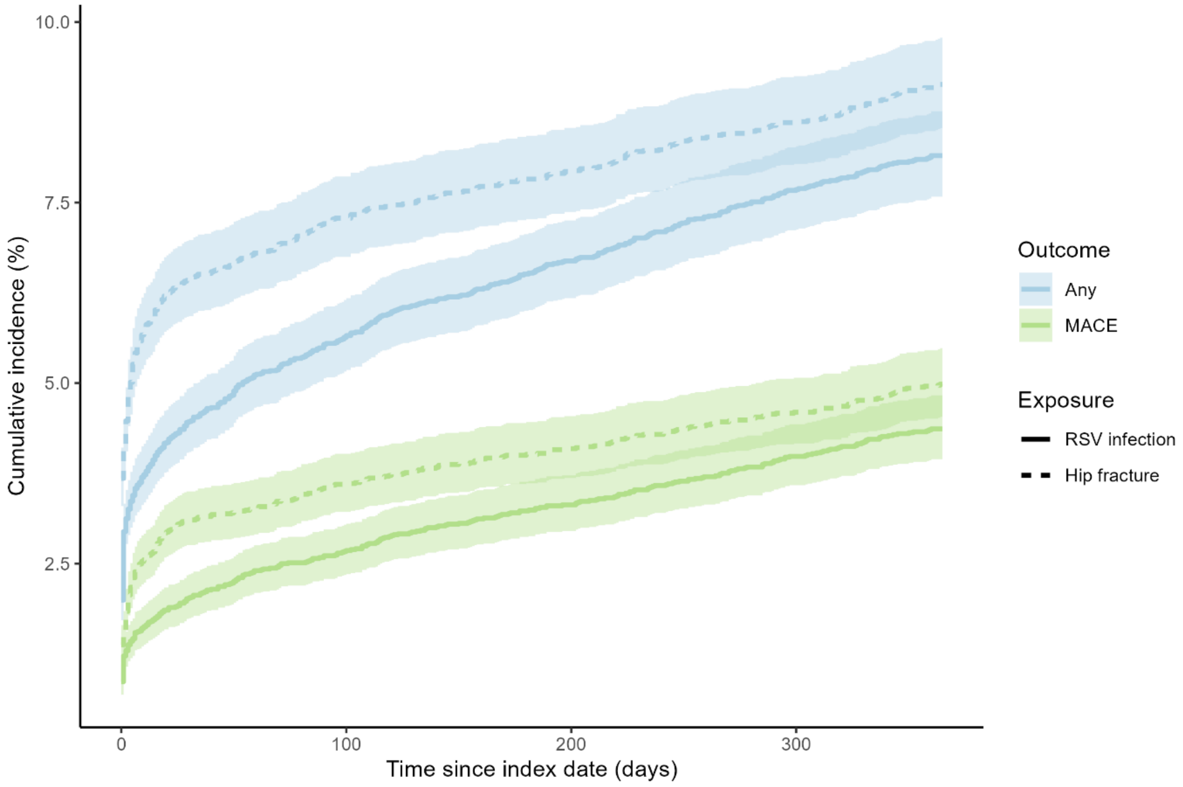

© 2025 Hviid A et al. *JAMA Network Open*.

eTable 4. Number of Individuals at Risk and Number of Any Cardiovascular Events and MACEs at Selected Time Points Among Individuals With RSV Infection and Individuals With Hip Fracture

			Time (days)						
			**0**	**10**	**20**	**30**	**100**	**200**	**365**
**Any**	RSV infected	*Events*	168	146	37	24	98	85	118
		At risk	8422	7717	7512	7398	7045	6736	5639
	Hip fracture	*Events*	310	172	42	17	69	51	89
		At risk	8422	7614	7344	7156	6565	5883	5054
**MACE**	RSV infected	*Events*	73	64	19	14	54	52	85
		At risk	8422	7879	7684	7577	7251	6953	5828
	Hip fracture	*Events*	116	99	32	14	40	39	66
		At risk	8422	7868	7594	7389	6800	6103	5252

© 2025 Hviid A et al. *JAMA Network Open*.

eFigure 5. Cumulative Incidences of Any Cardiovascular Event and MACE, Respectively, Among Individuals With RSV Infection and Individuals With Urinary Tract Infection Without Sepsis

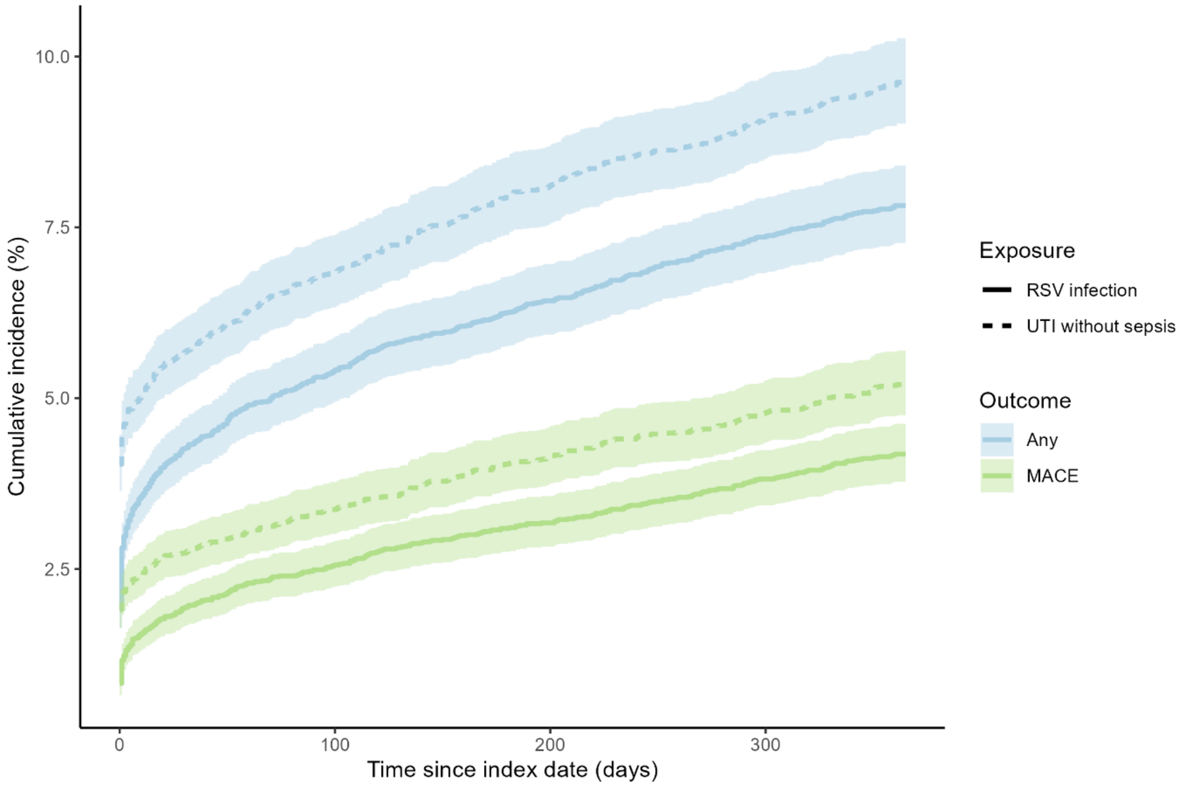

© 2025 Hviid A et al. *JAMA Network Open*.

eTable 5. Number of Individuals at Risk and Number of Any Cardiovascular Events and MACEs at Selected Time Points Among Individuals With RSV Infection and Individuals With Urinary Tract Infection Without Sepsis

			Time (days)						
			**0**	**10**	**20**	**30**	**100**	**200**	**365**
**Any**	RSV infected	*Events*	168	146	37	24	99	87	118
		At risk	8825	8109	7900	7786	7427	7115	5970
	Urinary tract infection w/o sepsis	*Events*	356	89	37	18	101	107	120
		At risk	8825	8266	8087	7952	7350	6673	5818
**MACE**	RSV infected	*Events*	73	64	19	14	54	53	85
		At risk	8825	8271	8072	7965	7634	7334	6160
	Urinary tract infection w/o sepsis	*Events*	168	45	22	6	55	66	83
		At risk	8825	8485	8311	8174	7576	6896	6029

© 2025 Hviid A et al. *JAMA Network Open*.
